# Plasma glucose, HbA1c, insulin and lipid profile in Sudanese type 2 diabetic patients with cardiovascular disease: a case control study

**DOI:** 10.12688/f1000research.110927.2

**Published:** 2024-10-07

**Authors:** Salsabbil Idris Abdallah, Nuha Eljailli Abubaker, Mariam Abbas Ibrahim, Ahmed Abd Alla, Rami Adam Humaida

**Affiliations:** 1 Clinical Chemistry Department, Sudan University of Science and Technology,, Khartoum, Khartoum, 11111, Sudan; 2 Department of Parasitology and Medical Entomology, Sudan University of Science and Technology, Khartoum, Khartoum, 11111, Sudan; 3 Department of lab, Modern medical center, Khartoum, Khartuom, 11111, Sudan

**Keywords:** FBS, HBA1c, T2DM, HDL-C, and HDL-C

## Abstract

**Background:** Type 2 diabetes mellitus (T2DM) and its consequences are a serious global public health issue. By 2030, the number of people with type 2 diabetes is predicted to reach 439 million. The purpose of this study is to evaluate the plasma levels of glucose, HbA1c, insulin, and lipid profile in Sudanese T2DM patients.

**Methods:** This case control study included 165 Sudanese patients with diabetic type 2 and a cardiovascular condition as cases and 165 diabetic type 2 volunteers without a cardiovascular disorder as controls. The concentrations of plasma glucose, HbA1c, and lipid profile were assessed using a Mindray BS-480 auto-chemistry analyzer, and insulin was analyzed using a Cobase 411 auto analyzer. The collected data were analyzed using statistical tools for social science computer programs (SPSS version 21).

**Results:** According to the findings, (59.4%) of patients between the ages of (50-69). Females made up 50.9%. (38.2%) of patients had an illness duration of between (8-15 years). (41.8%) of individuals did not have hypertension. There was a substantial rise in BMI, FBG, HbA1c, HDL-C, and insulin among diabetics with cardiovascular disease compared to diabetics without cardiovascular disease (p-value = 0.001, 0.000, 0.018, and 0.000). Females had significantly higher blood TC, LDL-C, HDL-C, and BMI than males (p-values = 0.000, 0,001, and 0.000, respectively). There were significant positive correlation between FBS, HBA1c, insulin and duration of disease (r=0.155, p, value=0.005) (r=0.160, p, value=0.004)(r=0.103, p. value=0.061)respectively, while there were significant negative correlation between TC, TG,LDL-C, HDL-C and duration of disease (r=-0.152, p, value= 0.006)(r=-0.023, p, value=0.678)(r=-0.113, p, value= 0.040)(r=-0.145, p, value=0.008)respectively.

**Conclusion:** When comparing diabetics with cardiovascular disease to diabetics without cardiovascular disease, there was a substantial rise in BMI, FBG, HbA1c, HDL-C, and insulin. FBS, HBA1c, insulin, and illness duration all had a strong positive connection.

## Introduction

T2DM has no established cause, however it is known to be caused by physiological, genetic, and environmental variables such as obesity, family history, and pollution ( Yang *et al.*, 2018). T2DM and its consequences are a major global public health issue, and cardiovascular disease (CVD) represents a significant health burden in Sub-Saharan Africa, including Sudan ( Almobarak *et al.*, 2018).

In fact, T2DM and CVD share several risk factors, such as obesity, hypertension, hyperglycemia, insulin resistance, serum lipid and lipoprotein abnormalities, which are defined mostly by high serum triglycerides and low levels of high-density lipoprotein (HDL) cholesterol ( Rosa *et al.*, 2018; Vepsäläinen, 2013) Hypertension in people with diabetes increases the risk of peripheral arterial disease, as well as dyslipidaemia, which is a significant risk factor for developing macrovascular complications in diabetic patients and contributes to an increased risk of coronary heart disease ( World Health Organization, 2006; Chokshi *et al.*, 2013). Diabetes, hypertension, and obesity or being overweight all raise the risk of cardiovascular problems and other morbidities ( Brown *et al.*, 2000). Insulin resistance, on the other hand, has been linked to a two to five-fold increase in CVD mortality and is a mixture of multiple cardiometabolic risk factors such as obesity, poor glucose metabolism, hypertension, and dyslipidemia. It is also a risk factor for type 2 diabetes ( Vepsäläinen, 2013). A recent study in Sudan found that low HDL is a common trait in two-thirds of diabetics, whereas high cholesterol and triglycerides were found in more than a quarter ( Awadalla *et al.*, 2018).

The evaluation of these lipid pathways and insulin resistance in Sudanese diabetes, in addition to the recurrent lipid profile study, may be an important cooperative step in the prediction of CVD since these atherogenic impacts may be mitigated by lifestyle changes. As a result, the purpose of this study was to evaluate the plasma levels of glucose, HbA1c, insulin, and lipid profile in Sudanese patients with T2DM.

## Methods

This analytical case control study was approved by the Clinical Chemistry Department and the Research Committee of the College of Medical Laboratory Science at Sudan University of Science and Technology, ethical approval No (DSR – IEC – 02 – 1 – 2020), a written informed consent was taken from all participants in the study for data collection and publication. Between March 2020 and August 2021, the research was conducted in the Zenam Diabetic Center and Sudan Cardic Center at Khartoum Siate. The case group consisted of 165 Sudanese patients with diabetes type 2 and a cardiovascular condition who were diagnosed by physicians, whereas the control group consisted of 165 diabetic type 2 volunteers who did not have a cardiovascular problem (the sample size was calculated by using sample size Formula n = p = the proportion to the target population. q = 1-p z = the standard normal deviation, usually set at 1.96, which correspond to the level 95% confidence level, d = degree of accuracy desired, set at 0.05). Patients with renal, liver, or thyroid disease, as well as those who smoked, drank, or had hepatitis A, B, or C, were excluded from the study as all that conditions may affect the levels of tested parameters. The risk factor criteria were Obesity, high blood pressure, high LDL or low HDL cholesterol levels, family history CVD and age.

The case and control groups were age matched, ranging from 30 to 100 years old. The demographic data was acquired via a questionnaire after all participants gave their informed permission. The heights and weights of all the participants were measured. The BMI was computed by dividing the weight (kg) by the square of the height (m ^2^) as proposed by Romero *et al.* (2012). Only one sample was obtained from each participant by using conventional vein puncture procedure, accordingly; 3 ml of blood was collected into a heparin container, spun at 3000 rpm for 3 minutes, and plasma was recovered and kept at -20°C until evaluated.

The concentrations of plasma glucose, HbA1c, and lipid profiles were determined using a Mindray BS-480 auto-chemistry analyzer, whereas insulin levels were determined using a Cobase 411 auto analyzer as proposed by Annani-Akollor *et al.* (2019). To ensure the accuracy and precision of the results, a level of control and calibrator given by the manufacturer in the reagent kit was evaluated using a sample run on a Mindary BS 480 auto analyzer.

### Statistical analysis

The information gathered was analyzed with the use of a computer application called statistical packages for social science (SPSS version 21). The results were reported as percentages with a mean and standard deviation; No data were missed. To compare means in groups and subgroups; the ANOVA test was employed, and Pearson's correlation test was utilized to establish the relationship between study variables. Statistical significance was defined as a p-value of less than 0.05.

## Results

In this study, 330 people were included, with 165 diabetes type 2 patients with cardiovascular disease as the case group and 165 diabetic type 2 patients without cardiovascular disease as the control group. The findings revealed that 11.5% aged 30 to 49 years, 59.4% of patients aged 50 to 69 years, and 29.1% of patients aged 70 to 100 years were in the 30 to 49 age group, 59.4% of patients aged 50 to 69 years, and 29.1% of patients aged 70 to 100 years. Females accounted for 50.9% of the total, while men accounted for 49.1%. Duration of DM (33.3%) ranges from one month to seven years, (38.2%) from eight to fifteen years, and (28.5%) from sixteen to twenty-five years (More than 15 years). HTN lasted 24.2% (from 1 month to 7 years), 17.6% of the time (8-15 years), 16.4% of the time (more than 15 years), and 41.8% of the time there was no HTN. When comparing diabetics with cardiovascular disease to diabetics without cardiovascular disease, there was a substantial rise in BMI (29.4 5.3 kg/m ^2^, p value = 0.001) as in Table 1.

**Table 1.** Demographic characteristic of individuals.

Variable	Diabetic with cardiovascular diseases	Diabetic without cardiovascular diseases
Age groups		
(30-49)	19 (11.5%)	60 (36.4%)
(50-69)	98 (59.4%)	82 (49.7%)
(70-100)	48 (29.1%)	23 (13.9%)
Gender		
Male	81 (49.1%)	71 (43.0%)
Female	84 (50.9%)	94 (57.0%)
Duration of DM (years)		
1 month – 7 years	55 (33.3%)	79 (47.9%)
8-15 years	63(38.2%)	50 (30.3%)
More than 15 years	47 (28.5%)	36 (21.8%)
Duration of HTN (years)		
1 month – 7 years	40 (24.2%)	25 (15.2%)
8-15 years	29 (17.6%)	12 (7.3%)
More than 15 years	27 (16.4%)	11 (6.7%)
No HTN	69 (41.8%)	117 (70.9%)
BMI	29.0 ± 3.4	27.4 ± 5.3
Family history of cardiovascular disease	10 (6.1%)	4 (2.4%)
No family history of cardiovascular disease	155 (93.9)	161 (97.6%)
Mean ± SD		(p = 0.001)

### Biochemical profile of individuals

The biochemical differences between people with and without cardiovascular disease shown in Table 2, there were significant increases in FBG, HBA1c, TG, HDL-c, and insulin between the two groups with p values 0.05, but no significant statistical difference in TC and LDL-c with p values > 0.05. (2).

**Table 2.** Mean concentrations of FBS, HBA1c TC, TG,HDL-C,LDL-C and insulin in case and control group.

Variable	Diabetics with cardiovascular diseases Mean ± SD	Diabetic without cardiovascular diseases Mean ± SD	p-value
FBS mg/dl	205 ± 73	178 ± 73	0.001
HB A1c	8.6 ± 2.4	7.5 ± 1.9	0.000
TC (mg/dl)	166 ± 52	173 ± 45	0.244
TG (mg/dl)	130 ± 77	112 ± 56	0.015
HDL-C (mg/dl)	41 ± 13	45 ± 11	0.018
LDL-C (mg/dl)	94 ± 37	101 ± 29	0.091
Insulin (mU/L)	22.9 ± 17.3	14.9 ± 14.0	0.000

As shown in Table 3, females had a significantly higher BMI, TC, LDL-C, and HDL-C than males, but FBG, HBA1c, TG, and insulin levels were not significantly different.

**Table 3.** Comparison of means of FBS, HBA1c TC, TG, HDL-C, LDL-C and insulin according to gender.

Variable	Male Mean ± SD	Female Mean ± SD	p-value
FBS (mg/dl)	196 ± 75	188 ± 73	0.321
HB A1c %	8.2 ± 2.1	7.9 ± 2.2	0.185
TC (mg/dl)	157 ± 40	181 ± 52	0.000
TG (mg/dl)	115 ± 59	126 ± 74	0.126
HDL-C (mg/dl)	40 ± 11	45 ± 13	0.000
LDL-C (mg/dl)	90 ± 30	103 ± 35	0.001
Insulin (mU/L)	19.3 ± 16.6	18.6 ± 15.8	0.704
BMI	26.9 ± 4.3	29.3 ± 4.4	0.000

### Correlation between biochemical profile and demographic features

The results indicated that HBA1candinsulin had a significant positive association with age, whereas LDL-c had a significant negative correlation with age. FBS, TC, TG, and HDL-c had no correlation with age. FBS, HBA1c, TC, TG, HDL-c, LDL-c, insulin, and BMI had no link, but there was a substantial positive correlation between FBS, HBA1c, and insulin and diabetes duration. As shown in Table 4, there was a strong negative association between TC, HDL-c, and LDL-c and diabetes duration, but no link between TG, insulin, and diabetes length.

**Table 4.** Correlations between Age, FBS, HBA1c TC, TG, HDL-C, LDL-C and insulin and study variables (Age, BMI, and duration of diabetes).

Correlation variables	r-value	p-value
Age and FBs	0.001	0.982
Age and HBA1c	0.115	0.037
Age and TC	- 0.068	0.220
Age and TG	0.044	0.427
Age and LDL-C	- 0.123	0.026
Age and HDL-C	-0.060	0.274
Age and insulin	0.122	0.027
BMI and FBs	0.005	0.931
BMI and HBA1c	0.022	0.696
BMI and TC	0.057	0.298
BMI and TG	0.019	0.735
BMI and LDL-C	0.052	0.348
BMI and HDL-C	0.060	0.278
BMI and insulin	0.021	0.699
Duration of DM and FBs	0.155	0.005
Duration of DM and HBA1c	0.160	0.004
Duration of DM and TC	-0.152	0.006
B Duration of DM and TG	-0.023	0.678
Duration of DM and LDL-C	-0.113	0.040
Duration of DM and HDL-C	-0.145	0.008
Duration of DM and insulin	0.103	0.061

## Discussion

The overall features of research participants revealed that patients aged 50-69 years old had the largest proportion of diabetics with cardiovascular disease. This finding was also obtained by Wu *et al.* (2015) who stated that the prevalence was greater among people aged 50 and older, as well as Teo and Dokainish (2017) reported that, the prevalence of CVD increased with age. Females had a larger prevalence of diabetic patients with cardiovascular complications (50.9%) than men (49.1 percent), this conclusion is similar to that of Teo and Dokainish (2017), which revealed a greater frequency of CVD in females.

The frequency of diabetic complications was much greater in individuals who had diabetes for a longer period of time. These findings are consistent with the findings of the Alam *et al.* (2021), which stated that long-term exposure to diabetes causes several diabetic problems. Furthermore, Rana *et al.* (2016) stated that patients with diabetes for more than ten years should be regarded to have a higher risk of cardiovascular disease.

This study found a significant difference in mean BMI, FBG, HBA1c, triglycerides, and insulin hormones between the two groups (P values 0.000, 0.001, 0.000, 0.015, and 0.000, respectively), as well as a significant decrease in mean HDL-C (P value 0.018), while there was no significant statistical difference in total cholesterol and LDL-C (P value > 0.05). Patients with T2DM at the LDL-C goal are nevertheless at a considerable risk of CVD events, according to a research by Timón *et al.* (2014) reported that, this residual risk is linked to a number of variables, including an increase in TG-rich proteins, a drop in HDL-C, and generalized obesity as measured by the body mass index (BMI), which is linked to a number of CVD risk factors and cardiovascular risk in T2DM patients.

This study found a significant increase in mean BMI, total cholesterol, HDL-C, and LDL-C in diabetic females when compared to diabetic males with a p value of 0.000. This finding is in line with another study that found: The prevalence of overweight in men and women varies depending on the country's level of development; higher BMI is more prevalent in men than women in high-income countries, whereas in low and middle-income countries, particularly in Arab countries, found a significant increase in mean BMI, total cholesterol, HDL-C, and LDL-C in women than men ( Aderibigbe *et al.*, 2018).

With a p value of > 0.05, there is no statistically significant difference in FBG, HBA1c, triglycerides, or insulin hormones. This finding is comparable to that of Moor *et al.* (2017) who found that mean total cholesterol and LDL-C levels were greater in women than in men, with a statistically significant difference, whereas HDL-C and triglycerides were not. This finding is also in line with that of Bertoluci and Rocha (2017) who found that women with diabetes had a greater chance of dying from cardiovascular causes than men.

Our research found a substantial positive link between HBA1c, insulin hormone levels, and diabetes patients' age (r = 0.115, p-value = 0.037, r = 0.122, p-value = 0.027). This result was verified by Huang *et al.* (2021) who found that there was a positive association between HBA1c level and age ( Huang *et al.*, 2021).

Although there was a significant negative association between LDL-C and age (r = -0.123, p = 0.026), there was no correlation between study parameters and BMI in this research. A significant positive correlation was also found between FBs, HBA1c level, and Duration of DM (r = 0.155, p-value = 0.005, r = 0.160, p-value = 0.004), as well as a significant negative correlation between total cholesterol, HDL-C, LDL-C, and Duration of DM (r = -0.152, p-value = 0.006, r = - 0.145, p-value = 0.040, r = -0.113, p = 0.040. This result is similar to another result from a study carried out by Priya and Begum (2020), which found a substantial positive link between HBA1c, total cholesterol, LDL-C, and duration of diabetes, but a significant negative correlation between HDL-C and duration of diabetes ( Priya and Begum, 2020).

### Study limitation

The samples were collected from two centers in Khartoum state.

## Data availability

Figshare. Salsabil. DOI: https://doi.org/10.6084/m9.figshare.19375664.v2 ( AbdAlla *et al.*, 2022)

Data are available under the terms of the Creative Commons Zero “No rights reserved” data waiver (CC BY 4.0 Public domain dedication).
