# Supplementary material for: Plasma glucose, HbA1c, insulin and lipid profile in Sudanese type 2 diabetic patients with cardiovascular disease: a case control study
Source: F1000Res. 2024 Oct 7;11:472. Originally published 2022 Apr 28. [Version 2] doi: 10.12688/f1000research.110927.2 (PMC11499864; doi:10.12688/f1000research.110927.2)
Supplement: Peer review history

# Reviewer report: Reviewer response for version 2

**DOI:** 10.5256/f1000research.172705.r329582

**Published:** 2024-10-23

**Authors:** Diarmuid Smith

**Affiliations:** 1 Beaumont Hospital/RCSI Medical School, Dublin, Ireland

I am happy with the changes made and happy for the article to be indexed.

Is the work clearly and accurately presented and does it cite the current literature?

Partly

If applicable, is the statistical analysis and its interpretation appropriate?

Partly

Are all the source data underlying the results available to ensure full reproducibility?

Partly

Is the study design appropriate and is the work technically sound?

Partly

Are the conclusions drawn adequately supported by the results?

Partly

Are sufficient details of methods and analysis provided to allow replication by others?

Partly

Reviewer Expertise:

Clinical diabetes care

I confirm that I have read this submission and believe that I have an appropriate level of expertise to confirm that it is of an acceptable scientific standard.

# Reviewer report: Reviewer response for version 2

**DOI:** 10.5256/f1000research.172705.r329581

**Published:** 2024-10-17

**Authors:** Jalal Taneera

**Affiliations:** 1 University of Sharjah, Sharjah, United Arab Emirates

I am happy with the revised version and do not have any further comments.

Is the work clearly and accurately presented and does it cite the current literature?

Yes

If applicable, is the statistical analysis and its interpretation appropriate?

Yes

Are all the source data underlying the results available to ensure full reproducibility?

Yes

Is the study design appropriate and is the work technically sound?

Yes

Are the conclusions drawn adequately supported by the results?

Yes

Are sufficient details of methods and analysis provided to allow replication by others?

Yes

Reviewer Expertise:

NA

I confirm that I have read this submission and believe that I have an appropriate level of expertise to confirm that it is of an acceptable scientific standard.

# Reviewer report: Reviewer response for version 2

**DOI:** 10.5256/f1000research.172705.r331686

**Published:** 2024-10-17

**Authors:** Zoran Gluvic

**Affiliations:** 1 University of Belgrade, Belgrade, Serbia

The authors tempted to evaluate the plasma levels of glucose, HbA1c, insulin, and lipid profile in Sudanese T2DM patients with cardiovascular diseases. The study is not the case control by design. English must be refined.

 Serious flaws must be addressed to improve the paper quality.

 1. The methods are questionable. The inclusion criteria must be clearly addressed. The use of ANOVA to assess the differences between two groups of subjects is not justified.

 2. The results are presented in helter-skelter manner. The tables should be fused to obtain better data visibility.

 3. The Discussion is very, very thin. The repeating of already mentioned results with no connection and discussion with already published results. No valid conclusions arise from the results, no future perspectives.

Is the work clearly and accurately presented and does it cite the current literature?

Partly

If applicable, is the statistical analysis and its interpretation appropriate?

Partly

Are all the source data underlying the results available to ensure full reproducibility?

Yes

Is the study design appropriate and is the work technically sound?

No

Are the conclusions drawn adequately supported by the results?

Partly

Are sufficient details of methods and analysis provided to allow replication by others?

Partly

Reviewer Expertise:

Diabetes, Thyroid diseases

I confirm that I have read this submission and believe that I have an appropriate level of expertise to state that I do not consider it to be of an acceptable scientific standard, for reasons outlined above.

# Reviewer report: Reviewer response for version 1

**DOI:** 10.5256/f1000research.122586.r179450

**Published:** 2023-06-20

**Authors:** Jalal Taneera

**Affiliations:** 1 University of Sharjah, Sharjah, United Arab Emirates

In this study, the authors assessed the plasma levels of glucose, HbA1c, insulin, and lipid profile in Sudanese T2DM patients. A case-control design was used, including 165 T2DM patients with cardiovascular conditions as cases and 165 T2DM patients without cardiovascular disorders as controls. Results showed a notable increase in BMI, fasting blood glucose (FBG), HbA1c, HDL cholesterol (HDL-C), and insulin in people with diabetes with cardiovascular disease. Females had higher TC, LDL-C, HDL-C, and BMI levels than males. Positive correlations were observed between FBS, HbA1c, insulin, and illness duration. These findings provide valuable insights into metabolic profiles and cardiovascular implications in Sudanese T2DM patients.

 The study is a straightforward investigation that can be improved by adding extra data (see comments below).

 Major Issues:

 The study will benefit from some English improvement.

 It was unclear in the study what was the criteria for cardiovascular complications .. author is requested to define such important issues.

 Family history of cardiovascular complications should be addressed in the T2DM patients with cardiovascular conditions group.

 It would be great if the authors showed the types of drugs described for these patients and subsequently dragged some data (if possible) if certain anti-diabetic drugs protect against cardiovascular complications compared to others.

 What are the clinical implications for the data? I failed to see any implications in the discussion.

 Please expand more on the study limitations.

 Other issues:

 HBA1c should be HbA1c.

 Define what is HTN.

Is the work clearly and accurately presented and does it cite the current literature?

Yes

If applicable, is the statistical analysis and its interpretation appropriate?

Yes

Are all the source data underlying the results available to ensure full reproducibility?

Yes

Is the study design appropriate and is the work technically sound?

Yes

Are the conclusions drawn adequately supported by the results?

Yes

Are sufficient details of methods and analysis provided to allow replication by others?

Yes

Reviewer Expertise:

Diabetes research

I confirm that I have read this submission and believe that I have an appropriate level of expertise to confirm that it is of an acceptable scientific standard, however I have significant reservations, as outlined above.

# Reviewer report: Reviewer response for version 1

**DOI:** 10.5256/f1000research.122586.r172786

**Published:** 2023-05-23

**Authors:** Diarmuid Smith

**Affiliations:** 1 Beaumont Hospital/RCSI Medical School, Dublin, Ireland

Thank you for asking me to review this article on cardiovascular disease in Sudanese patients with Type 2 diabetes.

 The article was interesting and describes CV risk in a Sudanese population with T2DM which I think would be interesting for your readers.

 However there are a number of issues with the paper.

 Major Issues 

1. The writing needs to be improved throughout the paper and there were a few paragraphs in which I could not understand what the author was trying to say for example page 3 paragraph 3 needs to be rewritten; page 3 the first paragraph of the results section needs to be rewritten as the results are not clear in this paragraph.
2. Throughout the manuscript the term diabetic with cardiovascular disease or diabetic needs to be changed to Type 2 diabetes with cardiovascular disease or Type 2 diabetes without cardiovascular disease
3. The paper would have been strengthened by having a non-diabetic control group with cardiovascular disease to see what the differences were in relation to BMI. lipids etc in those with and without diabetes
4. The data was collected in 2 centres in Khartoum. Were the lab tests done in the one lab or 2 separate labs and what was CV for the Aic assay and other assays? please comment
5. The authors ethical approval for the study was not obtained, please explain.
6. How was cardiovascular disease defined?
7. Why was smoking excluded?
8. Was there a difference in medication type between with and without cardiovascular disease particularly in relation to statin therapy and hypoglycaemic agents
9. Could the authors comment in the discussion section on why they thought the A1c was higher in those with CV disease compared to those without?
10. The term insulin hormone levels should be replaced with the term serum insulin levels.

I would be grateful if there manuscript could be rewritten to improve the content, understanding, results and discussion section of the paper.

Is the work clearly and accurately presented and does it cite the current literature?

Partly

If applicable, is the statistical analysis and its interpretation appropriate?

Partly

Are all the source data underlying the results available to ensure full reproducibility?

Partly

Is the study design appropriate and is the work technically sound?

Partly

Are the conclusions drawn adequately supported by the results?

Partly

Are sufficient details of methods and analysis provided to allow replication by others?

Partly

Reviewer Expertise:

Clinical diabetes care

I confirm that I have read this submission and believe that I have an appropriate level of expertise to confirm that it is of an acceptable scientific standard, however I have significant reservations, as outlined above.
